# Comparison of pedicle screw fixation by four different posterior approaches for the treatment of type A thoracolumbar fractures without neurologic injury

**DOI:** 10.3389/fsurg.2022.1036255

**Published:** 2023-01-05

**Authors:** Xu Zhu, Yijie Shao, Yun Lu, Jiajia Sun, Jie Chen

**Affiliations:** ^1^ Department of Orthopedics, The First Affiliated Hospital of Soochow University, Suzhou, China; ^2^ Orthopedic Institute, Soochow University, Suzhou, China

**Keywords:** thoracolumbar fractures, wiltse approach, pedicle screw, o-arm navigation, minimally invasive surgery, posterior approach

## Abstract

**Purpose:**

This study was designed to compare the pedicle screw fixation by four different posterior approaches for the treatment of type A thoracolumbar fractures without neurologic injury.

**Methods:**

A total of 165 patients with type A thoracolumbar fractures without neurologic injury who received pedicle screw fixation by posterior approaches from February 2017 to August 2018 were enrolled in this study. They were further divided into the following four groups according to different posterior approaches: Open-C group (conventional open approach), Open-W group (Wiltse approach), MIS-F group (percutaneous approach with fluoroscopy guidance), and MIS-O group (percutaneous approach with O-arm navigation). The demographic data, clinical outcomes, and radiologic parameters were evaluated and compared among the four groups.

**Results:**

There were no significant differences in age, gender, fracture segment, and follow-up time. The incision length, blood loss, hospital stay time, and VAS (Visual Analog Scale) and ODI (Oswestry Disability Index) scores at the early stage of post-operation were the worst in the Open-C group. The MIS-O group showed significantly higher accuracy rate of pedicle position than other groups. The preoperative and postoperative AVH (anterior vertebral height) and VWA (vertebral wedge angle) obtain obvious correction in all patients immediately after and 1 year post-operation. No difference was found among the four groups at the final radiographic follow-up.

**Conclusions:**

The four different posterior approaches are effective in treating type A thoracolumbar fractures in our study. Each approach has its own individual strengths and weaknesses and therefore requires comprehensive consideration prior to use. Proper approaches selection is critical to patients.

## Introduction

Thoracolumbar fractures are the foremost common fracture zone of spinal fractures which mainly involve the thoracolumbar junction (T10-L2) (1). The thoracolumbar junction is vulnerable to injury owing to its unique anatomic and biomechanical properties where almost 60% of spinal fractures occur (2, 3). According to AOspine classification, these fractures can be divided into three types: Type A (vertebral compression injury), Type B (tension band injury), and Type C (displacement or translational injury). The incidence of Type A thoracolumbar fractures is the highest among all types (1). Although the administration of thoracolumbar fractures without neurologic injury is still disputed, posterior pedicle screw fixation technique is used most frequently to restore vertebral body height and provide superior reconstruction stability (4).

Open approaches, including conventional open approach and Wiltse approach, remain a common method for thoracolumbar fractures. Conventional open posterior pedicle screw fixation approach remains universally adopted owing to its safety and short learning curve. However, the paraspinal muscle was stripped off widely *via* conventional open approach and many studies reported prolonged postoperative muscle pain (5, 6). To weak the muscle injury and related complications, Wiltse approach was used to expose the facet joints through the gap between the multifidus and longissimus. Li et al. found that Wiltse approach displayed lower multifidus muscle atrophy percentage and VAS score compared with conventional posterior open group (7).

Recently, minimally invasive surgery (MIS) has been rapidly developed and gradually became the preference on account of less injury and quicker recovery. Percutaneous pedicle screw fixation has gained popularity with less bleeding, less hospital stay, and less paraspinal muscle injury (8–10). Kocis et al. reported that percutaneous approach with fluoroscopy guidance resulted in higher radiation exposure dose compared with open approach (11). They also indicated that the percutaneous approach has no significant difference in fracture reduction compared with open approach. Controversially, several studies found that the open approach was related to better fracture reduction (12, 13). With the development of technology, the surgeon applies the percutaneous o-arm navigation pedicle screw in the clinic to enhance the accuracy of screw insertion. The pedicle screw is inserted in real time under 3D fluoroscopy of the O-arm navigation system. Yang et al. found O-arm assisted percutaneous screw fixation had advantages over conventional open approach screw fixation inaccuracy of pedicle screw placement (14). Lu et al. reported O-arm navigation could further enhance the accuracy, reduce facet joint violation and avoid pedicle perforation compared with fluoroscopy guidance for percutaneous pedicle screw fixation which was consistent with Yang's conclusion (15).

Although many studies have compared various clinical and radiological outcomes between different approaches for pedicle screw fixation, the agreement on the best choice for thoracolumbar fractures remained to be controversial due to each approach has its advantages and disadvantages (16–18). In addition, simultaneous comparison of four different approaches for posterior pedicle screw fixation seemed to be rarely reported. Our study aims to compare the clinical and radiological outcomes of four different posterior pedicle screw fixation approaches mentioned above for the administration of type A thoracolumbar fractures without neurologic injury.

## Materials and methods

### Patients

From February 2017 to August 2018, 165 patients with thoracolumbar fractures (T10-L2) without neurologic injury who were treated with pedicle screw fixation by posterior approaches were selected. This retrospective study was permitted by the Ethics Committee and Institutional Review Board of the First Affiliated Hospital of Soochow University. The inclusion criteria for this study were: (1) single-segment thoracolumbar fractures (T10-L2); (2) thoracolumbar fracture classified Type A according to AO Spine classification (1); (3) age ranged from 18 to 65; (4) the time from injury and operation was less than 10 days; (5) patient received pedicle screw fixation by one of the four different posterior approaches. Meanwhile, the exclusion criteria were as follows: (1) pathological fractures; (2) accompanied by nerve injury; (3) osteoporotic fractures; (4) spinal scoliosis and ankylosing spondylitis. Based on pedicle screw fixation by different approaches, then the patients following criteria were divided into four groups: Open-C group (*n* = 48, conventional open approach), Open-W group (*n* = 35, Wiltse approach), MIS-F group (*n* = 39, percutaneous approach with fluoroscopy guidance), and MIS-O group (*n* = 43, percutaneous approach with O-arm navigation). The follow-up time was at least 12 months postoperatively. The demographic data of the enrolled patients were listed in Table 1.

**Table 1:** Demographic data of the four groups.

Demographics	Open-C	Open-W	MIS-F	MIS-O	*P*-value
Patient number	48	35	39	43	
Age (years)	45.3 ± 9.0	46.3 ± 10.8	48.8 ± 12.8	45.8 ± 9.2	*P* = 0.674
Gender					*P* = 0.105
Female	18	9	14	7	
Male	30	26	25	36	
Fracture segment					*P* = 0.982
T10	2	2	3	4	
T11	2	3	4	4	
T12	13	10	7	9	
L1	23	14	17	18	
L2	8	6	8	8	
Follow-up (months)	15.8 ± 1.7	15.4 ± 2.0	15.9 ± 2.1	16.3 ± 1.5	*P* = 0.139

Open-C, conventional open approach; Open-W, Wiltse approach; MIS-F, percutaneous approach with fluoroscopy guidance; MIS-O, percutaneous approach with O-arm navigation.

### Surgical procedures

All surgeries in this study were performed by the same chief physician. All patients were prone after general anesthesia. In the open groups (including Open-C and Open-W), an about 8-centimeter posterior midline incision was cut centered on the fracture segment. We exposed the facet joints *via* stripping the paraspinal muscle from the spinous process and lamina by conventional open approach. Differently, we exposed the facet joints *via* the gap between the multifidus and longissimus by Wiltse approach. The entry point of pedicle was determined based on bony landmarks, located at the junction of the lateral margin of the superior articular process and the transverse process. In the MIS groups (including MIS-F and MIS-O), four small paramedian incisions were made. We inserted the pedicle screw *via* real-time fluoroscopic visualization by percutaneous approach with fluoroscopy guidance. For the O-arm navigation assistance, after fixing the reference frame on the spinous process, we inserted the pedicle screw with the help of the Stealth Station navigation system (Medtronic Sofamor Danek). Then we again performed 3D scan with O-arm to verify the placement of pedicle screw. Two bent rods were implanted and the vertebral body height was corrected by distraction. No drainage was installed.

### Clinical evaluation

The length of incision, blood loss, operation duration, intraoperative radiation dose, length of hospital stays, hospitalization costs, and postoperative walking time were compared among the four groups. The length of incision in MIS group is the sum of the four small incision lengths. The Visual Analog Scale (VAS) and the Oswestry Disability Index (ODI) scores were assessed preoperation, 3 days, 1 month, 6 months, and 12 months post-operation to reflect the quality of daily life. The clinical evaluation was performed by physicians unrelated to the surgical procedures.

### Radiologic evaluation

**Figure 1:**
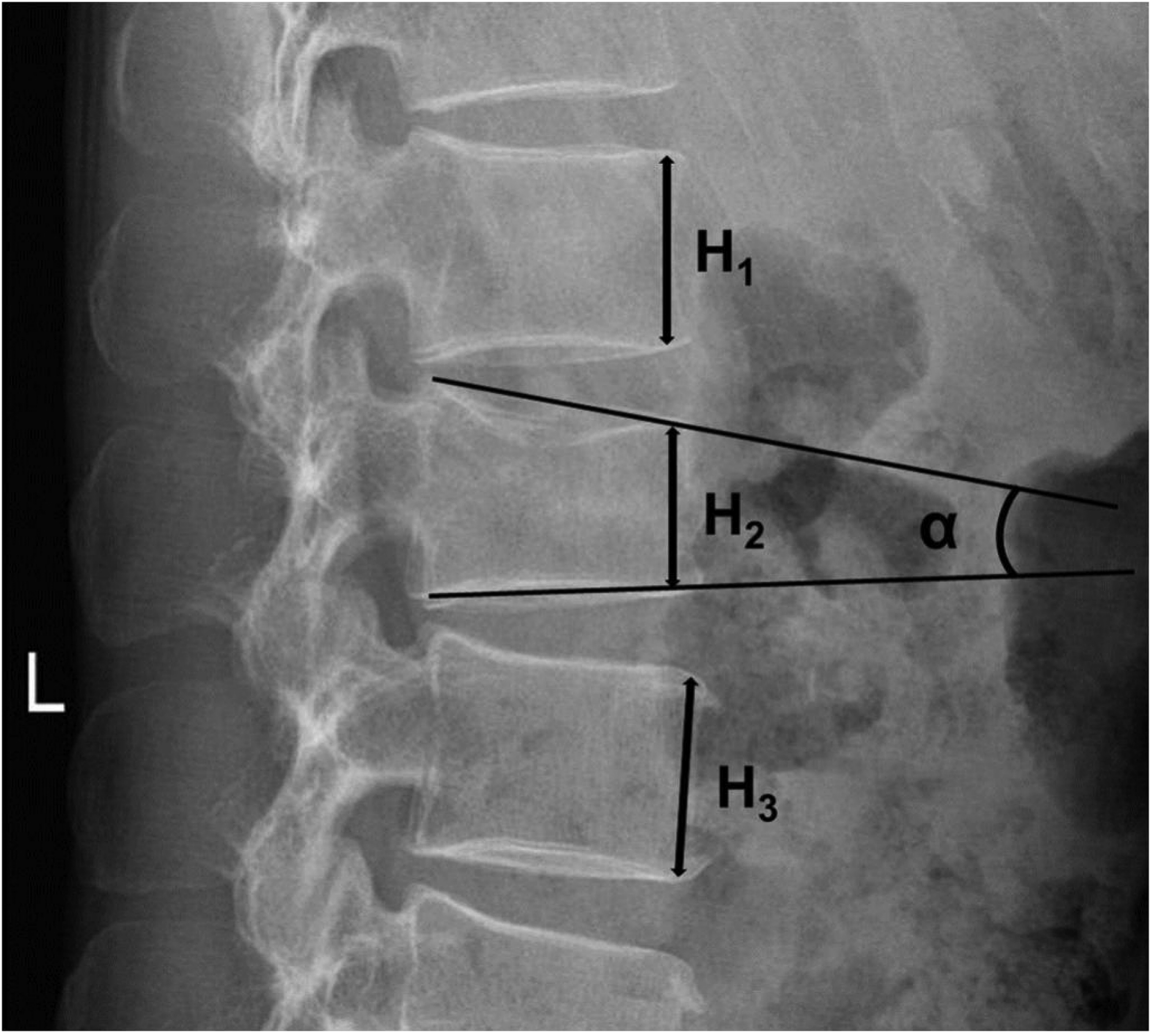
Schematic diagram of radiological parameters measurement of the four groups. H_1_: the anterior height of the vertebrae above the injured level; H_2_: the anterior height of the injured vertebrae; H_3_: the anterior height of the vertebrae below the injured level. AVH(%) = 2H_2_/(H_1 _+ H_3_); VWA(°):*α*, the angle between the superior endplate and inferior endplate of the injured vertebrae.

The anterior vertebral height (AVH, %) and vertebral wedge angle (VWA, °) were measured on lateral x-rays. The pedicle screw placement accuracy was assessed based on postoperative CT images according to the previous study (19). AVH was defined as the fractured vertebrae's anterior height divided by the anterior mean height of the vertebrae above and below the injured level (Figure 1). VWA was defined as the angle between the upper endplate and lower endplate of the fractured vertebrae (Figure 1). The radiologic evaluation was performed by physicians unrelated to the surgical procedures.

### Statistical analysis

All analyses were performed with Sigmaplot 14.0 (SystatSoftware, Inc). Continuous variables were described as mean ± standard deviation. The differences in continuous variables were calculated with one-way ANOVA analysis and the following Tukey's test for multiple comparisons. The chi-square test was used to compare the gender and fracture segment distribution among the four groups. *P* value < 0.05 was considered statistically significant for all tests.

## Results

A total of 165 patients were enrolled in this study, including 48 females and 117 males. The mean age and follow-up time were 46.5 ± 10.5 years and 15.9 ± 1.8 months, respectively. The demographic data of the four groups were shown in Table 1. There were no significant differences in age, gender, fracture segment, and follow-up time among the four groups (*P* > 0.05). There were no complications including nail break, screw withdrawal, and screw loosening during the follow-up.

**Figure 2:**
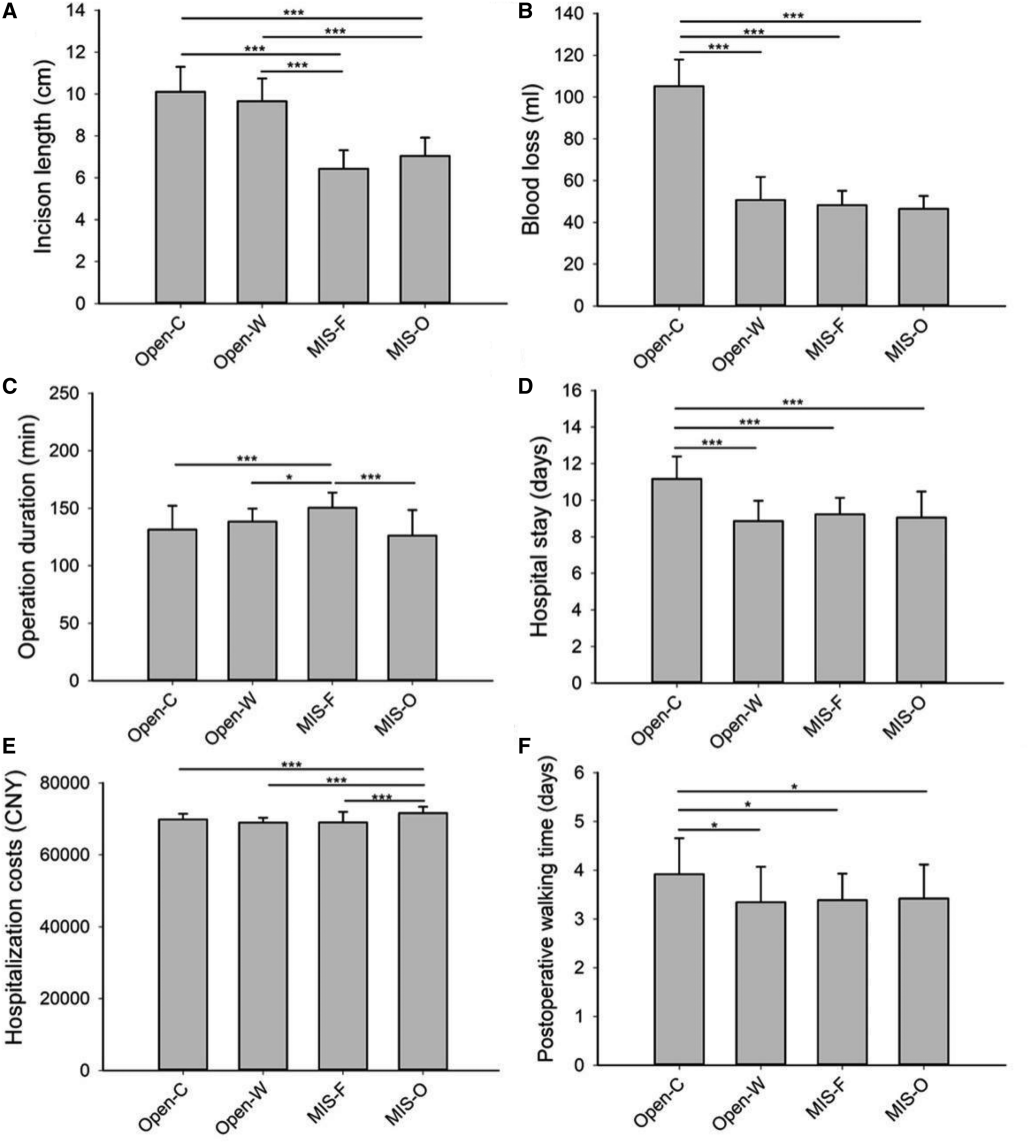
Comparison of various clinical parameters among the four groups. Comparison of various clinical parameters including incison length (**A**), blood loss (**B**), operation duration (**C**), hospital stay (**D**), hospitalization costs (**E**) and postoperative walking time (**F**) among the four groups. Open-C, conventional open approach; Open-W, Wiltse approach; MIS-F, percutaneous approach with fluoroscopy guidance; MIS-O, percutaneous approach with O-arm navigation. *P < 0.05; **P < 0.01; ***P < 0.001.

The detailed clinical parameters of the four groups were listed in Table 2. The incision length was 10.1 ± 1.2 cm in the Open-C group and 9.7 ± 1.1 cm in the Open-W groups, which was significantly longer than that in the other two groups (*P* < 0.001) (Figure 2A). The blood loss was 105.2 ± 12.6 ml in the Open-C group and was significantly higher in the Open-C group than that in the other three groups (*P* < 0.001) (Figure 2B). Similarly, the operation duration was 150.4 ± 13.1 min in the MIS-F group which was the highest among the four groups (Figure 2C). The hospital stay and postoperative walking time in the Open-C group were statistically longer than those in the other three groups (*P* < 0.05) (Figures 2D,F). In addition, the hospitalization costs in the MIS-O group were the highest among the four groups (*P* < 0.001) (Figure 2E).

**Table 2:** Clinical outcomes of the four groups.

Clinical parameters	Open-C	Open-W	MIS-F	MIS-O	*P*-value
Incision length (cm)	10.1 ± 1.2	9.7 ± 1.1	6.4 ± 0.9	7.0 ± 0.9	*P* < 0.001
Blood loss (ml)	105.2 ± 12.6	50.7 ± 11.0	48.3 ± 6.7	46.5 ± 6.1	*P* < 0.001
Operation duration (min)	131.3 ± 20.6	138.4 ± 11.2	150.4 ± 13.1	126.2 ± 22.0	*P* < 0.001
Hospital stay (days)	11.2 ± 1.2	8.9 ± 1.1	9.2 ± 0.9	9.0 ± 1.4	*P* < 0.001
Hospitalization costs (CNY)	69,851.7 ± 1566.6	68,966.0±1311.6	69,011.3±2901.2	73,632.8±2141.1	*P* < 0.001
Postoperative walking time (days)	3.9 ± 0.7	3.3 ± 0.7	3.4 ± 0.5	3.4 ± 0.7	*P* < 0.001

Open-C, conventional open approach; Open-W, Wiltse approach; MIS-F, percutaneous approach with fluoroscopy guidance; MIS-O, percutaneous approach with O-arm navigation.

**Figure 3:**
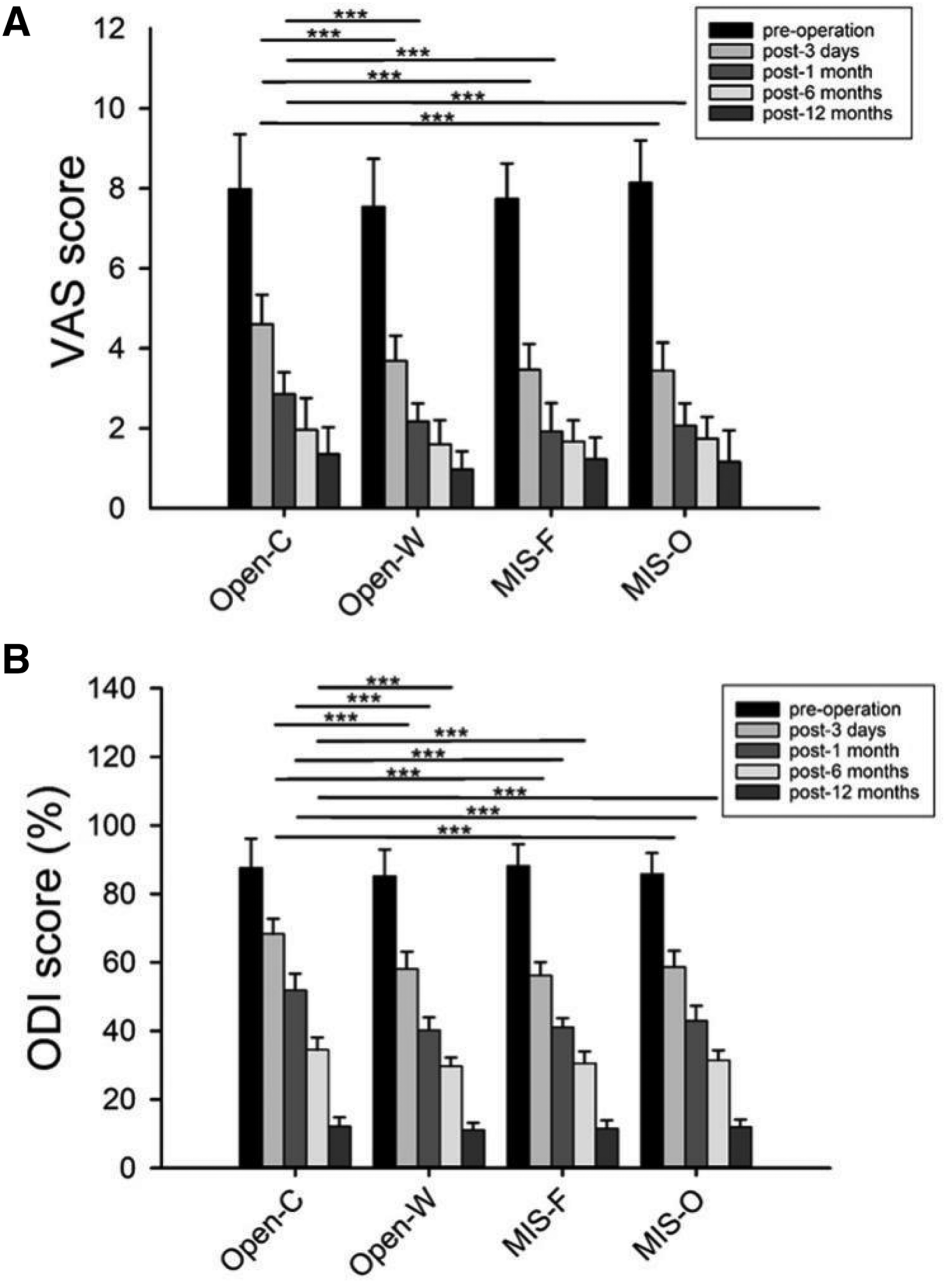
Comparison of VAS and ODI scores before operation and at different follow-up times among the four groups. Comparison of VAS (**A**) and ODI (**B**) scores before operation and at different follow-up times among the four groups. VAS, Visual Analogue Scale; ODI, Oswestry Disability Index; Open-C, conventional open approach; Open-W, Wiltse approach; MIS-F, percutaneous approach with fluoroscopy guidance; MIS-O, percutaneous approach with O-arm navigation. *P < 0.05; **P < 0.01; ***P < 0.001.

The VAS and ODI scores pre-operation and at different follow-up times of the four groups were documented in detail in Table 3. There were no statistical differences in preoperative VAS (*P* = 0.178) and preoperative ODI scores (*P* = 0.320) among the four groups. With the increase of follow-up time, the VAS and ODI scores in individual groups reduced gradually. The VAS and ODI scores in the Open-C groups 3 days after operation were significantly higher than those in the other three groups (*P* < 0.001) (Figures 3A,B). The VAS and ODI scores in the Open-C groups 1 month after operation were 2.9 ± 0.5 and 51.9 ± 4.8, which were the highest among the four groups (*P* < 0.001) (Figures 3A,B). Besides, the ODI score in Open-C group 6 months after operation was significantly higher than that in the other three groups (*P* < 0.001). However, there was no statistical difference in VAS score 6 months after operation among the four groups (*P* = 0.145). 12 months after operation, no differences were found in both VAS and ODI scores among the four groups (*P* = 0.074 for VAS and *P* = 0.290 for ODI score).

**Table 3:** VAS and ODI scores before operation and at different follow-up times of the four groups.

Parameters	Open-C	Open-W	MIS-F	MIS-O	*P*-value
VAS					
Pre-operation	8.0 ± 1.4	7.5 ± 1.2	7.7 ± 0.9	8.1 ± 1.0	*P* = 0.178
Post-3 days	4.6 ± 0.7	3.7 ± 0.5	3.5 ± 0.6	3.4 ± 0.7	*P* < 0.001
Post-1 month	2.9 ± 0.5	2.2 ± 0.4	1.9 ± 0.7	2.1 ± 0.5	*P* < 0.001
Post-6 months	2.0 ± 0.8	1.6 ± 0.6	1.7 ± 0.5	1.7 ± 0.5	*P* = 0.145
Post-12 months	1.4 ± 0.7	1.0 ± 0.4	1.2 ± 0.5	1.2 ± 0.8	*P* = 0.074
ODI (%)					
Pre-operation	87.7 ± 8.4	85.2 ± 7.7	88.2 ± 6.2	85.9 ± 6.0	*P* = 0.320
Post-3 days	68.3 ± 4.4	58.1 ± 5.0	56.2 ± 3.8	58.6 ± 4.7	*P* < 0.001
Post-1 month	51.9 ± 4.8	40.2 ± 3.7	41.0 ± 2.7	43.0 ± 4.3	*P* < 0.001
Post-6 months	34.5 ± 3.6	29.7 ± 2.5	30.6 ± 3.4	31.4 ± 2.9	*P* < 0.001
Post-12 months	12.2 ± 2.6	11.1 ± 2.0	11.5 ± 2.3	12.0 ± 2.1	*P* = 0.290

VAS, Visual Analogue Scale; ODI, Oswestry Disability Index; Open-C, conventional open approach; Open-W, Wiltse approach; MIS-F, percutaneous approach with fluoroscopy guidance; MIS-O, percutaneous approach with O-arm navigation.

The radiographic outcome before operation and at different follow-up times of the four groups was documented in detail in Table 4. Table 4 shows that the anterior vertebral height and kyphosis angle postoperatively has been achieved significant correction vs. preoperatively (*P* < 0.05). There was no significant difference among the groups in the AVH, VWA, and the correction loss of them at the final follow-up (*P* > 0.05). As shown in Table 4, the screw position (Grades 0) was statistically higher accuracy with O-arm navigation than that in the other groups (163/172, 94.7% vs. 162/192, 84.3% vs. 119/140, 85.7% vs. 128/156, 82.1%, respectively; *P* < 0.01).

**Table 4:** Radiologic parameters including AVH, VWA and accuracy of pedicle screw placement of the four groups.

Radiologic parameters	Open-C	Open-W	MIS-F	MIS-O	*P-*value
AVH (%)					
Pre-operation	68.1 ± 10.3	63.7 ± 10.9	67.8 ± 9.8	70.8 ± 11.5	*P* = 0.218
Post-operation	97.1 ± 6.2*	96.2 ± 5.4*	96.9 ± 6.6*	96.1 ± 6.6*	*P* < 0.05
Final follow-up	93.2 ± 8.1	90.3 ± 7.4	93.9 ± 5.1	92.1 ± 10.1	*P* = 0.371
Correction loss	4.1 ± 5.9	5.9 ± 6.4	3.1 ± 4.2	4.0 ± 7.3	*P* = 0.152
VWA (°)					
Pre-operation	14.3 ± 7.9	15.6 ± 3.6	12.2 ± 4.2	13.8 ± 4.7	*P* = 0.213
Post-operation	2.8 ± 3.4*	3.9 ± 2.4*	2.1 ± 2.2*	4.8 ± 3.3*	*P* < 0.05
Final follow-up	3.6 ± 3.7	4.7 ± 1.3	2.8 ± 2.9	5.5 ± 3.5	*P* = 0.312
Correction loss	0.8 ± 1.9	0.8 ± 1.2	0.7 ± 1.3	0.7 ± 2.3	*P* = 0.098
The accuracy rate of	162/192	119/140	128/156	163/172	*P* < 0.01
Pedicle screw placement (%)	84.3%**	85.7%**	82.1%**	94.7%	

AVH, The anterior vertebral height; VWA, The injured vertebral angles; Open-C, conventional open approach; Open-W, Wiltse approach; MIS-F, percutaneous approach with fluoroscopy guidance; MIS-O, percutaneous approach with O-arm navigation.

*P < 0.05 compared with Preopratively; **P < 0.01 compared with MIS-O group.

## Discussion

The thoracolumbar fractures are the common site of spine fracture encountered in clinic because of the spinal biomechanical characteristics. Numerous studies have shown nonoperative administration is effective for most thoracolumbar spine fracture (2), however treatment of thoracolumbar fractures remain controversial. A novel new Thoracolumbar Injury Classification and Severity Score (TLISS) have been proposed to guide the clinical decision of surgeon (20, 21). Some studies indicated significant fracture angle (>10°) increase or worse pain symptoms as an indication for consideration of surgery (22). A variety of posterior approaches have been used, including the conventional open approach, Wiltse approach, percutaneous approach with fluoroscopy guidance, and with O-arm navigation for thoracolumbar fractures. Each approach has its unique strengths and learning curve. In the current study, we systematically compared the surgical efficacies concerning demographic data, clinical outcomes, and radiologic parameters among these approaches.

Conventional open approach is a widely popular surgical technique due to its safety and short learning curve. Nevertheless, Wild et al. have revealed a series of disadvantages about traditional open surgery, including iatrogenic muscle injury, soft tissue injury, and denervation, which may result in weak muscle strength and backache (23). In our current study, incision length, blood loss, and length of stay were the worst in Open-C group, which is corroborated by several published studies (10, 12). Figure 3 showed there was no significant difference in the VAS and ODI scores postoperative 12 months among the four groups while the VAS and ODI scores in the Open-C groups at the early-stage of post-operation were statistically the highest. Similar to the previous study, postoperative walking time in the Open-C group was statistically worse than those in the other three groups, and these findings of our study indicated conventional open approach has a slightly positive impact on their early-stage postoperative recovery compared to other approaches (10).

To avoid these disadvantages, the Wiltse approach was first used to treat lumbar spine fractures in 1968 (24). Compared with conventional open approach, the Wiltse approach had the benefits of being less blood loss, period of hospitalization, and postoperative walking time except for incision length, ensuring earlier recovery. As minimally invasive surgery develops, percutaneous pedicle screw fixation was initially reported in the treatment of the lumbar spine in 1984 by Magerl (25). Our study shows percutaneous pedicle screw insertion is associated with a shorter incision length compared to the Open and Wiltse approaches. Fan et.al. stated that percutaneous and Wiltse approaches could avoid unnecessary paraspinal muscles injuries, and drastically reduce perioperative complications and back pain (9, 16). This study also shows that patients treated with percutaneous approach have less blood loss, shorter hospital stay, and postoperative walking time than those in Open-C group. However, the MIS group has no significant differences in the above three parameters, the VAS and ODI scores compared with Open-W group except for incision length, suggesting that the two approaches were similarly effective for thoracolumbar fractures. And the current study found the operation time of the MIS-F group was the longest among four groups. It is associated with manipulating C-arm multiple times during operation, further leading to higher doses of radiation. Furthermore, minimally invasive surgery has a steeper learning curve and surgeon's experience should not be neglected (26).

To ensure the safety of patients, the accuracy of pedicle screws placement has been a critical concern in spine surgery. Although the conventional C-arm system has facilitated the accuracy of pedicle screws placement for most patients, it deserve further effort for patients with distorted positions or scoliosis. With evolving imaging technology, O-arm real-time 3D navigation was introduced to direct surgeons to percutaneously insert pedicle screws. O-arm navigation provided a promising option for this kind of patient. Following our results, the MIS-O group showed a statistically higher accuracy rate of pedicle position compared to other groups (163/172, 94.7%), which is consistent with previous study (15). Several studies have indicated O-arm navigation had significant benefits in minimizing radiation and offering minimally invasive technique advantages compared with open approach (14, 15). Many studies have pointed out that O-arm guidance also has specific elements, including looseness of locator, register error of devices, and the micro deformations of the tracer, that would result in the misplacement of pedicle screws (15). Navigation coupled with O-arm imaging has a specific learning curve compared to other approaches (14). To sum up, O-arm navigation-related transcutaneous pedicle instrumentation offers a better therapy in the treatment of the specific patient.

The preoperative and postoperative AVH and VWA obtain obvious correction in all patients immediately after and 1 year postoperation, nevertheless no difference was found among four groups at the final radiographic follow-up (12, 14). Similarly, Palmisani et al. pointed out that there were no statistical differences in lumbar spine fracture between percutaneous and traditional fixation in terms of AVH and VWA (27, 28). Moreover, Sun et al. indicated that the radiological outcome in MIS and Open-W groups were better than Open-C group in correction loss (9). However, the Open-C group was advantageous over percutaneous approach in postoperative radiographic correction. The less spinal soft tissue damage avoided destroying the integrity of ligamental structure in MIS and Open-W groups, which reduced the correction loss (9).

To our knowledge, this is the first study that evaluates the possible advantages of these surgical approaches for type A thoracolumbar fractures. There are still several limitations in our study. First, only single-segment thoracolumbar fractures were analyzed in our study, and the surgical efficacies among the four different posterior approaches were still unclear for multiple-segment fractures. Second, the follow-up period was relatively short (mean follow-up period, 15.85 months). Third, the present study was a retrospective study, increasing the risk of patient selection bias. Future prospective studies need to include more patients and longer follow-up periods. Meanwhile, the treatment of multiple-segment thoracolumbar fractures will be further analyzed in the future study.

In conclusion, the four different posterior approaches are effective in treating type A thoracolumbar fractures in our study. Each approach has its own individual strengths and weaknesses and therefore requires comprehensive consideration prior to use. We think the surgical approach choice is based on the clinical characteristics of patients and the surgical experience of surgeon. Proper approaches selection is critical to patients.

## Data Availability

The raw data supporting the conclusions of this article will be made available by the authors, without undue reservation.
